# The Use of Psychedelics in the Treatment of Medical Conditions: An Analysis of Currently Registered Psychedelics Studies in the American Drug Trial Registry

**DOI:** 10.7759/cureus.29167

**Published:** 2022-09-14

**Authors:** Joshua S Kurtz, Neal A Patel, Julian L Gendreau, Chenyi Yang, Nolan Brown, Nick Bui, Bryce Picton, Mark Harris, Matthew Hatter, Ryan Beyer, Ronald Sahyouni, Luis Daniel Diaz-Aguilar, Joel Castellanos, Nathaniel Schuster, Mickey E Abraham

**Affiliations:** 1 Neurological Surgery, University of California Irvine, Irvine, USA; 2 Neurological Surgery, Mercer University School of Medicine, Savannah, USA; 3 Biomedical Engineering, Johns Hopkins University, Baltimore, USA; 4 Neurological Surgery, Loma Linda School of Medicine, Loma Linda, USA; 5 Neurological Surgery, University of California Irvine (UCI) School of Medicine, Irvine, USA; 6 Neurological Surgery, UCI, School of Medicine,, Irvine, USA; 7 Neurological Surgery, University of California San Diego, San Diego, USA; 8 Anesthesiology and Reanimation, University of California San Diego, San Diego, USA; 9 Center for Pain Medicine, University of California San Diego, San Diego, USA

**Keywords:** psilocybin, post traumatic stress disorder, mdma, major depressive disorder, clinical trials

## Abstract

Although early therapeutic research on psychedelics dates back to the 1940s, this field of investigation was met with many cultural and legal challenges in the 1970s. Over the past two decades, clinical trials using psychedelics have resumed. Therefore, the goal of this study was to (1) better characterize the recent uptrend in psychedelics in clinical trials and (2) identify areas where potentially new clinical trials could be initiated to help in the treatment of widely prevalent medical disorders. A systematic search was conducted on the clinicaltrials.gov database for all registered clinical trials examining the use of psychedelic drugs and was both qualitatively and quantitatively assessed. Analysis of recent studies registered in clinicaltrials.gov was performed using Pearson’s correlation coefficient testing. Statistical analysis and visualization were performed using R software. In totality, 105 clinical trials met this study’s inclusion criteria. The recent uptrend in registered clinical trials studying psychedelics (p = 0.002) was similar to the uptrend in total registered clinical trials in the registry (p < 0.001). All trials took place from 2007 to 2020, with 77.1% of studies starting in 2017 or later. A majority of clinical trials were in phase 1 (53.3%) or phase 2 (25.7%). Common disorders treated include substance addiction, post-traumatic stress disorder, and major depressive disorder. Potential research gaps include studying psychedelics as a potential option for symptomatic treatment during opioid tapering. There appears to be a recent uptrend in registered clinical trials studying psychedelics, which is similar to the recent increase in overall trials registered. Potentially, more studies could be performed to evaluate the potential of psychedelics for symptomatic treatment during opioid tapering and depression refractory to selective serotonin reuptake inhibitors.

## Introduction and background

Although hallucinogens have been used as spiritual tools for millennia in non-Western cultures [1,2], they were not introduced into the Western scientific community until 1896 when Arthur Heffter, a German pharmacologist, isolated mescaline from peyote [3]. After this period in time, the study of psychedelics became much more robust throughout the mid-1900s with the work of Albert Hofmann who studied the psychoactive properties of LSD (lysergic acid diethylamide) and psilocybin [4,5]. Researchers began to study the potential therapeutic uses of psychedelics for depression, alcoholism, and palliative care. LSD became a model psychedelic for these therapeutic developments [6,7]. Ultimately, tens of thousands of patients were treated in the 1950s and 1960s, predominantly in the psychotherapy setting [8,9]. Despite minimal adverse events [10], most psychedelics were later criminalized and deemed schedule 1 drugs by the United Nations Convention on Psychotropic Substances in 1971 due to their hypothesized close association with cultural turmoil and anti-Vietnam war politics of this period. These stringent regulations stigmatized psychedelic research, leaving investigators discouraged in the wake of these rapid changes [11]. Additionally, some authors argue that the decline of research into psychedelics was more a result of the difficulty to establish efficacy of the psychedelics given their mechanism of action and its clash with controlled trial methodologies at the time [12].

In the last two decades, there has been a resurgence of psychedelics research that has broadly encompassed the fields of neuroimaging [13-21], psychopharmacology [18,22-24], and psychology [25-31]. Focused mostly on psilocybin and MDMA (methyl​enedioxy​methamphetamine), researchers are now considering the potential of these drugs being used to treat a variety of different psychiatric and neurological conditions such as addiction, pain, depression, end-of-life anxiety, and post-traumatic stress disorder (PTSD). For example, one study published in 2016 by Roland Griffiths and his team at Johns Hopkins was a randomized, double-blind, crossover trial. This study gave cancer patients with poor prognoses and associated anxiety/depression either a high dose of psilocybin or a low dose, functioning as a placebo [32]. Results showed decreases in both clinician- and self-rated measures of depressed mood and anxiety among the participants in the high-dose group, along with a general increase in quality of life.

Cannabis, and specifically tetrahydracannibidinol (THC), also plays a major role in this field of research. A recent report published by the National Academies of Sciences, Engineering, and Medicine provided a comprehensive review of over 20 years of cannabis research, considering more than 10,000 scientific abstracts [33]. In this report, a committee discussed the health impacts of cannabis and cannabis-derived products, ranging from therapeutic properties to increased risks for causing cancers, respiratory diseases, and psychological disorders.

Despite the promising results that these investigations have yielded, there are still many barriers to advancing psychedelics research. Stigma, legality, and cultural interest all influence the amount of research that can be conducted in any field, but is especially prominent in the area of psychedelics. Ultimately, the history of psychedelics all but requires promising results to be accepted with cautious optimism, leaving researchers, clinicians, and the general public alike urging for a greater body of research into the therapy and safety of these drugs. The purpose of this study is to review the current scope/character of current psychedelic drug clinical trials, identify current cultural/legal challenges hindering progress in this field, and discuss potential avenues for future investigation.

## Review

Methods

This analysis of clinical trials studying psychedelic drugs was conducted using the ClinicalTrials.gov database, a database that is supported by the National Library of Medicine through the National Institutes of Health (Bethesda, Maryland, USA). This database contains more than 380,000 research studies conducted throughout the United States and in 220 countries. This database can be accessed at https://clinicaltrials.gov/. Information about trials is submitted by the sponsor or lead investigator for the purposes of research integrity by establishing prespecified primary outcomes. In addition, this registration of clinical trials also ensures publication of negative or null findings in addition to positive findings. This database is continually updated as the study progresses while updating the number of participants and preliminary results.

The authors queried the database using the input “psychedelic” in the “other terms” parameter. The search was made on May 1, 2021. Of the studies identified, those that had been suspended, terminated, withdrawn, or otherwise unknown statuses were excluded. For the studies that met the inclusion criteria, the following data were extracted: identifier number, title, recruitment status, condition or disease, study type, intervention, primary purpose, clinical phase, estimated number of participants, year of study initiation, country of origin, and sponsoring institution. Studies including cannabidiol (CBD) and kratom were excluded from the study.

Descriptive statistics were used for the initial summary of the retrieved data. Statistical analysis was performed with R software (R Core Team, Vienna, Austria) using the Pearson’s correlation test to discover if there were any uptrends in clinical trials with each successive year included in the study. This was performed for both the total number of clinical trials established on the clinicaltrials.gov website and to the clinical trials of psychedelics retrieved from the search. A p-value of 0.05 was used for establishing statistical significance, in addition to 95% confidence intervals. When analyzing for increasing trends in clinical trials, the year 2020 was omitted due to the reduced amount of medical research as a result of the COVID-19 pandemic. Visualization was performed using R software.

Results

**Figure 1:**
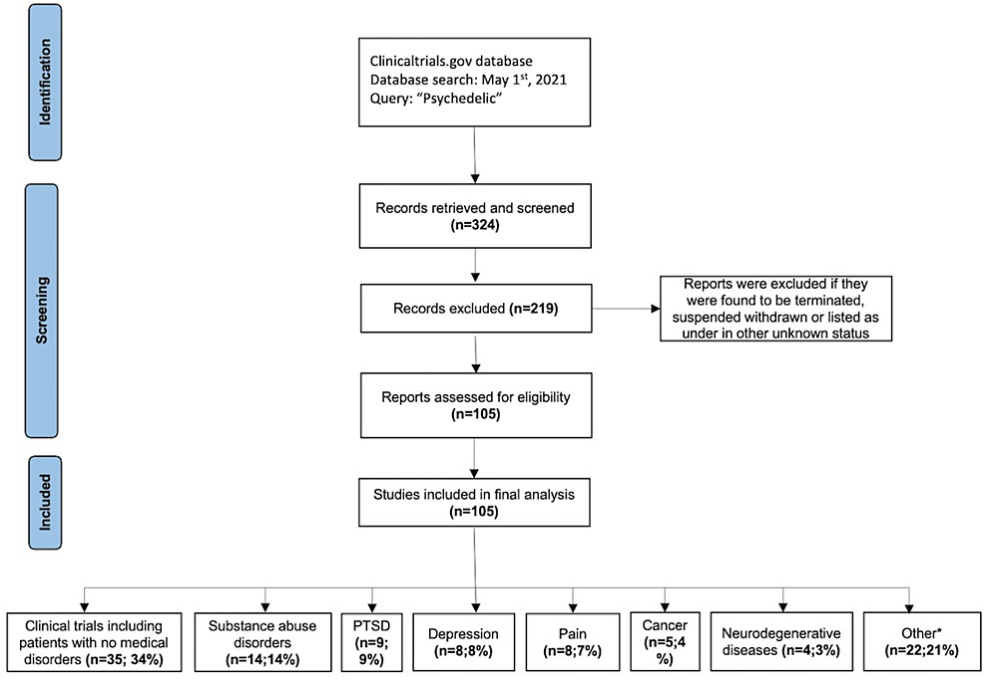
Results of the clinical trials search strategy. Flowchart depicts the search and screening process used to identify relevant clinical trials. PTSD, post-traumatic stress disorder

The search results included 105 studies that met this study’s inclusion criteria (Appendix A). A flowsheet of the inclusion/exclusion criteria is depicted in Figure 1. In total, 103 studies (98.1%) were interventional and two (1.9%) were observational, including one (1%) cross-sectional study and one (1%) prospective study. All trials took place from 2007 to 2020, with 81 (77.1%) studies starting in 2017 or later. Sixty-one trials had an enrollment between 0 and 50 participants (57%), 24 had a sample size between 51 and 100 participants (22.4%), 19 had a sample size between 101 and 500 participants (17.8%), and one had a sample size of >501 participants (0.9%). The mean number of study participants was 117 in all trials. No trials were completed. However, 19 (18.1%) were active, 63 (60%) were recruiting, one (1%) was enrolling through invitation, and 22 (21%) were not yet recruiting.

Country of Origin and Sponsoring Institutions

**Figure 2:**
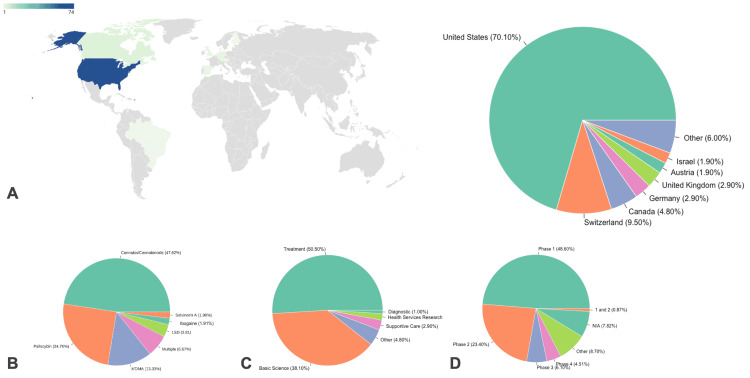
Characteristics of included clinical trials. (A) Clinical trials by nationality. Other includes clinical trials in the countries of Brazil, Denmark, Finland, Netherlands, Spain, and the West Indies. (B) Psychedelic drugs under analysis in each clinical trial. (C) Type of clinical trial. (D) Stage of currently reported clinical trials underway. 1 and 2 refer to trials including patients in both phase 1 and phase 2, respectively. N/A refers to trials with no listed phase. Other refers to exploratory trials before phase 1.

The United States of America has the most clinical trials, 74 (70.5%), with the rest originating in Switzerland (9.5%), Canada (4.8%), and several other countries (Table 1; Figure 2A). These studies were largely sponsored by Yale University (21.9%, n = 23), followed by Johns Hopkins University (10.5%, n = 11) and the Multidisciplinary Association for Psychedelic Studies (MAPS) (8.6%, n = 9).

**Table 1:** Characteristics of clinical trials included in this analysis

Characteristic	Number of trials	Percentage of all trials
Primary purpose		
Treatment	53	50.50%
Basic science	40	38.10%
Other	5	4.80%
Supportive care	3	2.90%
Health services research	2	1.90%
Diagnostic	1	1.00%
Phase		
1	56	53.30%
2	27	25.70%
3	7	6.70%
4	5	4.80%
Other	10	9.50%
N/A	9	8.60%
1 and 2	1	1.00%
Country of origin		
United States	74	70.50%
Switzerland	10	9.50%
Canada	5	4.80%
Germany	3	2.90%
United Kingdom	3	2.90%
Austria	2	1.90%
Israel	2	1.90%
Brazil	1	1.00%
Denmark	1	1.00%
Finland	1	1.00%
The Netherlands	1	1.00%
Spain	1	1.00%
West Indies	1	1.00%

Types of Psychedelics

The most commonly studies psychedelics were cannabinoids (47.62%, n = 50), and psilocybin (24.76%, n = 26). MDMA was also used (13.33%, n = 14). Other less common psychedelics were also studied including LSD, ibogaine hydrochloride, and salvinorin A (Figure 2B).

Purpose of Included Clinical Trials

The primary purposes of these trials were based on the following: treatment (50.5%, n = 53), basic science (38.1%, n = 40), other/unspecified (4.8%, n = 5), supportive care (2.9%, n = 3), health services research (1.9%, n = 2), and diagnostic (1.0%, n = 1) (Figure 2C).

Phases of Included Clinical Trials

The majority of the clinical trials are in phase 1 (53.3%, n = 56) or phase 2 (25.7%, n = 27). In addition, three studies are in phase 3 (2.9%) and five (4.8%) studies are in phase 4. An overview of study characteristics is depicted in Figure 2D.

Statistical Analysis

**Figure 3:**
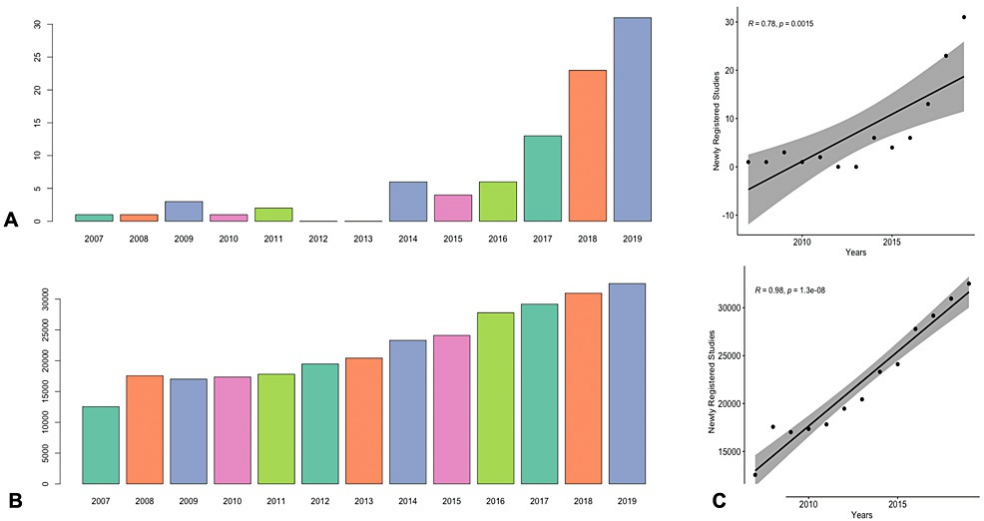
Amount of both psychedelic clinical trials and total clinical trials in the clinicaltrials.gov registry have increased over time. (A) Bar plot depicting the number of newly registered psychedelic-specific studies each year. (B) Bar plot depicting the number of newly registered studies in the clinicaltrials.gov database overall each year. (C) Pearson correlation analysis finding statistically significant association of increasing psychedelic studies per year in addition to overall registered trials in the clinicaltrials.gov registry.

Both the number of clinical trials specifically measuring psychedelics and the number of trials in the overall registry were found to be increasing over time (Figures 3A, 3B). Pearson’s correlation testing revealed an uptrend with an increasing number of psychedelic clinical trials occurring each year from 2007 to 2019 (r = 0.784 [95% CI: 0.411-0.932], p = 0.002). The resulting t-test statistic value was 4.192. In addition, there was also an increase in the total number of registered clinical trials each year as an entirety (r = 0.98 [95% CI: 0.919-0.993], p < 0.01). The resulting t-test statistic value was 14.832 (Figure 3C).

Condition or Disease

**Figure 4:**
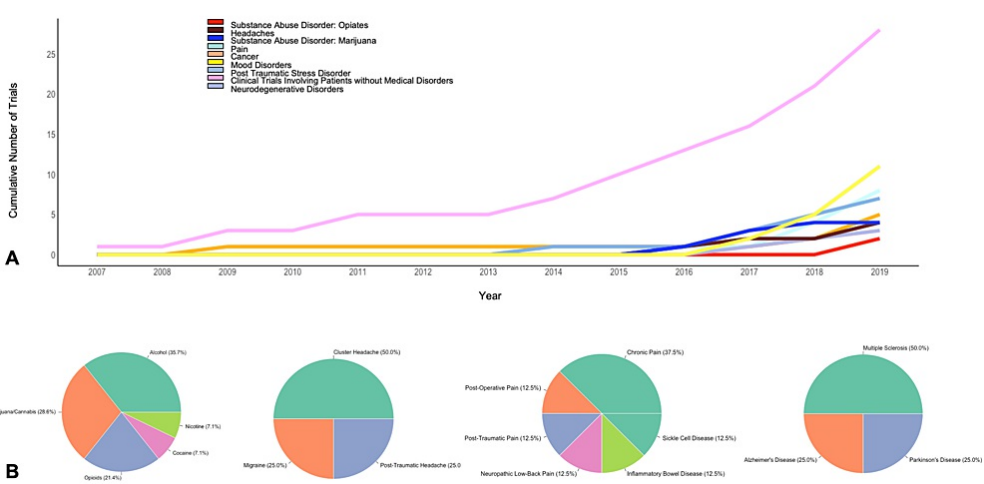
Number of psychedelic clinical trials per treating condition. (A) Line plot depicting cumulative number of clinical trials over time stratified by medical disorders. (B) Pie charts depicting percentage of psychedelic studies in the treatment of substance abuse disorder, headaches, pain, and neurodegenerative disorders.

Healthy participants made up the largest group, who were studied in 35 (33.3%) trials, with 30 of them being phase 1 trials (Table 2). The most commonly studied disorders were substance use disorders, with 14 studies (13.3%). More specifically, there were five alcohol, four marijuana/cannabis, three opioids, one cocaine, and one nicotine clinical trial regarding substance use. PTSD and depression were the next most frequently studied disorders, having nine (8.6%) and eight (7.6%) clinical trials, respectively. Eight clinical trials were conducted regarding pain, with chronic pain as the most studied (2.9%, n = 3). Studies regarding cancer/cancer-related symptoms accounted for five of the results. Degenerative disorders, consisting of multiple sclerosis, Alzheimer’s disease, Parkinson’s disease, and mild cognitive impairment had a total of four (3.7%) clinical trials. Headache disorders had a total of four (3.7%) clinical trials included as well. Three (2.8%) studies were included about psychosis/schizophrenia. There were also four trials where multiple conditions or diseases were studied, most commonly with depression and related disorders (Figures 4A, 4B).

**Table 2:** Number of clinical trials by condition or disease. PTSD, post-traumatic stress disorder; OCD, obsessive-compulsive disorder; HIV, human immunodeficiency virus

Condition or disease	Number of trials	Percentage of all trials
Healthy	35	33.3%
Substance use disorder	14	13.3%
Alcohol	5	4.8%
Marijuana/cannabis	4	3.8%
Opioids	3	2.9%
Cocaine	1	1.0%
Nicotine	1	1.0%
PTSD	9	8.6%
Depression	8	7.6%
Pain	8	7.6%
Chronic pain	3	2.9%
Post-operative pain	1	1.0%
Post-traumatic pain	1	1.0%
Neuropathic low back pain	1	1.0%
Inflammatory bowel disease	1	1.0%
Sickle cell disease	1	1.0%
Cancer	5	4.8%
Degenerative diseases	4	3.8%
Multiple sclerosis	2	1.9%
Alzheimer's disease	1	1.0%
Parkinson's disease	1	1.0%
Headache disorders	4	3.8%
Cluster headache	2	1.9%
Migraine	1	1.0%
Post-traumatic headache	1	1.0%
Multiple conditions or diseases	4	3.8%
Depression, Anxiety, PTSD	1	1.0%
Depression, depressive symptoms, Alzheimer's disease, mild cognitive impairment	1	1.0%
Distress/grief, depression	1	1.0%
Tourette syndrome, tic disorder	1	1.0%
Psychosis/schizophrenia	3	2.9%
OCD	2	1.9%
Anorexia nervosa	1	1.0%
Anxiety disorders	1	1.0%
Autism spectrum disorder	1	1.0%
Bipolar disorder	1	1.0%
Hepatic impairment	1	1.0%
HIV	1	1.0%
Obstructive sleep apnea	1	1.0%
Tourette syndrome	1	1.0%
Trichotillomania	1	1.0%

Study Interventions

Nearly half of all clinical trials were conducted with cannabis/cannabinoids as the study intervention (47.6%, n = 50) (Table 3). Dronabinol, a synthetic substance containing compounds from the cannabis plant, was used in 23 of those studies. The interventions consisted of psilocybin, MDMA, and LSD in 26 (24.8%), 14 (13.3%), and four (3.8%) studies, respectively. There were seven clinical trials that investigated all or a combination of psychedelic substances, and in these psilocybin was most commonly administered alongside other drugs (n = 3).

**Table 3:** Number of clinical trials by intervention. THC, tetrahydracannibidinol; CBD, cannabidiol; THC, tetrahydracannibidinol; PEA, palmitoylethanolamide; MDMA, methyl​enedioxy​methamphetamine; LSD, lysergic acid diethylamide; SSRI, selective serotonin reuptake inhibitors

Study intervention	Number of trials	Percentage of trials
Cannabis/cannabinoids	50	47.6%
Dronabinol	23	21.9%
THC	12	11.4%
Dronabinol/CBD	3	2.9%
THC/CBD	3	2.9%
Nabilone	2	1.9%
Nabiximols	2	1.9%
THC/terpenes (alpha-pinene, limonene)	2	1.9%
THX-110 (dronabinol + PEA)	2	1.9%
Inje cocktail, THC cannabis extract, THC/CBD cannabis extract	1	1.0%
Psilocybin	26	24.8%
MDMA	14	13.3%
Multiple interventions	7	6.7%
All psychedelics	1	1.0%
MDMA, methamphetamine	1	1.0%
Psilocybin, ketamine	1	1.0%
Psilocybin, LSD	1	1.0%
Psilocybin, SSRI (escitalopram)	1	1.0%
THC, ketamine	1	1.0%
Dronabinol, ethanol	1	1.0%
LSD	4	3.8%
Ibogaine hydrochloride	2	1.9%
Salvinorin A	2	1.9%

Discussion

The sheer number of recently established current clinical trials reveals that research is increasing in this area, especially since 2017 [34]. Statistical analysis of all trials registered into the database, however, suggests an increasing amount of studies in all fields. Thus, the increasing trend in psychedelic intervention studies could represent just improved registration of clinical trials overall by all medical researchers.

Furthermore, it is evident that many of these studies are still in their infancy. Many researchers are still facing the challenge of first establishing the safety of hallucinogenic drugs. Thus, the fact that more than 50% of the current trials are phase 1 is not surprising. This is especially imperative as the schedule 1 status of many psychedelic substances requires researchers to firmly establish the safety of the drugs on healthy subjects before moving on to their potential therapeutic impacts.

Conditions or Diseases

With the exceptionally heavy burden of addiction and overdose rates in the United States [34], and in the wake of the current opioid epidemic, it was fitting that substance use disorders overall were the most commonly studied conditions in current psychedelic clinical trials. However, only three trials focused specifically on opioid substance use disorder. Two of the studies are testing psychedelics as maintenance therapy along with buprenorphine/naloxone, while one clinical trial is testing psychedelics as an adjunct with methadone withdrawal. This is a large gap in this field of research as approximately 70,000 Americans suffered from overdoses causing fatalities in 2018, and two-thirds of those were from opioids [35,36]. Thus, successful treatments in this realm of psychedelic research could elicit a substantial impact on a psychiatric disorder with rapidly increasing prevalence and rates of mortality. Ibogaine, a naturally occurring alkaloid for which there are two clinical trials underway, has shown promise in reducing alcohol and opioid cravings and withdrawals; however, its application is limited by its hallucinogenic and arrhythmogenic adverse effects [37]. Cameron et al. recently formulated an analog, tabernanthalog (TBG), that addresses both issues, which sets it apart as a candidate for substance use disorder clinical trials [3].

The next most studied disorders are PTSD and major depressive disorder, as both have nine and eight studies currently underway, respectively. There is already early evidence that psychedelic treatment could be successful in treating these disorders [36-39]. It is important to consider that the term “post-traumatic stress disorder (PTSD)” was also not a term before its first appearance in 1980, when it was initially described in the third edition of the Diagnostic and Statistical Manual of Mental Disorders. Therefore, this could potentially have affected our search, thus not identifying a significant number of trials before the term became incorporated into mainstream use and study. However, trauma-based research could potentially have still been studied and registered using different terminologies and descriptions. The present study's results on MDD trial prevalence align with Carhart-Harris and Goodwin, who, in a review outlining the therapeutic potential of psychedelics, accept that treatment-resistant depression is the most logical place to focus inquiry given the uncertainty in the treatment plan after SSRI failure [6]. The future should ideally focus on creating innovative therapies for patients with SSRI refractory disease. There are also non-psychiatric disorders that are currently being studied. Four studies used psychedelics to treat neurodegenerative disorders and eight studies evaluated treatment options for different forms of pain (chronic, post-operative, post-traumatic). Twelve studies were also found measuring psychedelic use for treating pain and headache disorders, while eight studies specifically evaluated psychedelic use for pain (chronic, postoperative, post-traumatic) and four studies evaluated treatment of headache disorders.

Interventions

The most common substances used in interventional studies were cannabis/cannabinoids. A large number of drugs (dronabinol, nabilone, nabiximols, THX-110) fell under this category as there are multiple synthetic cannabinoids currently under development or already brought to market. The fact that most studies use dronabinol is understandable seeing as it is already FDA-approved for appetite stimulation and as an antiemetic to combat chemo-induced nausea and vomiting, while nabilone is FDA-approved for nausea and vomiting refractory to conventional medical management [40-41]. With that in mind, current trials are studying broader uses of these drugs as treatments for chronic pain, Alzheimer’s, sleep apnea, and PTSD.

The next most researched drug is psilocybin, with 26 studies underway. This is the most popular drug of the “classical psychedelics” in clinical trials. This is due to the promising research that has already been performed with psilocybin and, historically, with a drug that has similar subjective effects, LSD (which is itself currently being researched in four different studies). Furthermore, there is an expert consensus that these two drugs cause less harm to society and individuals alike as compared to alcohol, tobacco, and other recreational drugs [42-45]. The success of previous studies is clear from the exciting FDA breakthrough therapy designation that it received in both 2018 and 2019, a promising pattern in the context of being a schedule I drug with "no currently accepted medical use" [46]. Current studies are focused on psychiatric issues such as depression, OCD, and alcohol-use disorder, but they are also studying potential uses in treating headaches and anorexia. The sheer number of studies is a promising sign that the preliminary success of prior studies is being taken seriously and being further advanced.

MDMA is the next most studied substance, with 14 studies ongoing. This reflects previous success in studies researching MDMA-assisted psychotherapy as a treatment for PTSD. The first controlled clinical trial of MDMA-assisted psychotherapy was published in 2011 [47] and produced promising results as 83% of the experimental group no longer met criteria for PTSD at 2- and 12-month follow-ups. There have subsequently been further promising studies in this area of research, and MDMA-assisted psychotherapy was even granted a breakthrough therapy designation from the FDA in 2017. It is important to continue to push for more robust clinical trials with high-quality randomized design and appropriate blinding. Additionally, researchers should aim for large enough sample sizes to ensure adequate power of detecting treatment effects that are not due to chance alone.

Primary Purpose and Phases

Although there has been a renewed and inspired interest in psychedelic therapies, the use of psychedelics overall is ultimately still in a nascent stage. This is reflected in the fact that only 53 out of 105 studies are studying the substances as treatments (rather than, for example, basic science research) and that 84/92 studies that are subject to classical study phases are in either phase 1 or phase 2 trials with only three trials in phase 3 and five in phase 4. Of the classical psychedelics (MDMA, LSD, psilocybin, ibogaine) there are only two current trials in phase 3 and they are both studying MDMA treatments for PTSD. This is also limited by financial constraints as both phase 3 and phase 4 clinical trials require more financial backing and are generally funded by industry. Potentially, when more studies progress, there will be an exponential increase in both the volume and speed of the research.

Geography

The United States of America has the most clinical trials out of any country, with 74 studies currently underway. This is despite strict government regulations regarding schedule 1 drugs and is a promising sign that regulations may loosen in the coming years.

The country with the most studies on a per-capital basis, however, is Switzerland, with 10. This may be reflective of the power of stigma and culture in facilitating research. Switzerland has a long history of being more accepting of psychedelic use, even legalizing LSD and MDMA therapies from 1988 to 1993 [48] and granting individual allowances for the therapeutic use of LSD and MDMA since 2014 [49]. It is no surprise that the country that celebrates “bicycle day” is also the country with the highest rate of research on the topic [50]. This holiday became commemorated after Albert Hofmann first synthesized and intentionally self-ingested LSD. Thus, he experienced the effects of the substance while also riding on a bicycle. This was one of the first well-known events where the hallucinogenic properties of LSD were identified, and thus Switzerland is now regarded largely as the birthplace of LSD.

It is also important to consider that several other clinical trial databases exist such as the European clinical trials registry at https://www.clinical trialsregister.eu. In addition, an Australian registry can be found at https://www.australianclinicaltrials.gov.au. This study only analyzed registered characteristics of each trial found on the clinicaltrials.gov website, which most likely created a bias toward being predominantly U.S. clinical trials compared to the other data from the other databases.

Types of Clinical Trials

When reviewing the clinical trial questions and their hypotheses over time, it appears that the questions asked by researchers have become more robust after each consecutive year. The few initial trials before 2010 mainly consisted of using psychedelics for the treatment of mood symptoms such as after cancer treatment (NCT00957359), during smoking cessation (NCT01943994), and for psychological therapy (NCT01404754). These studies had the primary goal of improving the mood of patients that underwent separate treatments for their medical diseases. Now recently, psychedelics are being used to actually treat many diseases as the sole drug of choice including many psychiatric diseases. Additionally, many studies have now also been added to achieve even basic scientific pursuits. Randomization with quadruple masking of the participant, care provider, investigator, and outcomes assessor appeared largely throughout all the years of clinical trials for psychedelics.

Current Challenges

While adverse psychotic reactions could theoretically be adverse events of psychedelic treatments, there has so far been an absence of any such reaction in recent studies [8,50,51]. Indeed, researchers now consider hallucinogens as one of the classes of drugs with the least amount of adverse side-effects [43,52-55]. Most countries have scheduled psychedelic drugs, increasing the standards of research design needed to approve and conduct research with them [40].However, there remain many factors that limit the potential application of psychedelics in a clinical setting. These barriers, compounded with a lack of acceptance from mainstream medicine and weariness from the general public, urge psychedelic researchers to adopt a measured approach if progress is to be achieved [56,57]. As a result, many current trials are small-scale, early phase studies to observe the safety and tolerability of this class of drugs [58]. Ultimately, if there is to be progress, it will likely be slow, which is not unwelcome by the psychedelic community. However, overcoming this image will not just depend upon sound research, as there are early data that suggest the therapeutic effects of psychedelics are correlated with the degree of the subjective opinion on the efficacy of the drugs. Thus, the progression of the field with research studies may potentially be hindered once again due to stigma. Ultimately, a proactive approach to performing rigorous research is needed for future innovation in the field. This could be potentially performed by obtaining a better basic science understanding of the drugs on a molecular level and educating the public. In addition, educating the public on the safety profile of these drugs is paramount.

Limitations

This study has several limitations. Only one United States sponsored database was searched. The opinions of the patients in these trials could also not be evaluated, and future studies should examine patient attitudes toward these particular drugs as treatment options for their medical disorders.

## Conclusions

In the past two decades, there has been a recent uptrend in clinical trials of psychedelic drugs. Psychedelic therapies potentially hold much promise for the treatment of psychiatric disorders, but their current legal status and social stigmatization will likely continue to be a barrier to their progression to becoming a widely used treatment option for patients. However, the progress that has occurred over the years is encouraging and shows that the field is trending positively. More studies could be performed to evaluate the potential of psychedelics for symptomatic treatment during opioid tapering and depression refractory to selective serotonin reuptake inhibitors. Ultimately, a proactive approach to educating the scientific and general community alike is warranted.

## Appendices

**Table 4:** Supplemental A. Total data of clinical trials in response to the query of clinicaltrials.gov. CBD, cannabidiol; HIV, human immunodeficiency virus; LSD, lysergic acid diethylamide; MDMA, methyl​enedioxy​methamphetamine; OCD, obsessive compulsive disorder; PEA, palmitoylethanolamide; SSRI, selective serotonin reuptake inhibitors; THC, tetrahydracannibidinol

Clinical trials number	Title	Recruitment status	Condition or disease	Study type	Intervention	Primary purpose	Phase	Estimated enrollment	Study start date	Country	Sponsor
NCT03984214	Efficacy and Safety of Dronabinol in the Improvement of Chemotherapy-induced and Tumor-related Symptoms in Advanced Pancreatic Cancer	Recruiting	Cancer	Interventional	Cannabis/cannabinoids – dronabinol	Treatment	3	140	2019	Austria	Austrian Group Medical Tumor Therapy
NCT04003948	Preliminary Efficacy and Safety of Ibogaine in the Treatment of Methadone Detoxification	Not yet recruiting	Substance use disorder – opioids	Interventional	Ibogaine hydrochloride	Treatment	2	20	2019	Spain	Barcelona
NCT03756974	BX-1 in Spasticity Due to Multiple Sclerosis	Recruiting	Degenerative diseases – multiple sclerosis	Interventional	Cannabis/cannabinoids – dronabinol	Treatment	3	384	2019	Germany	Bionorica SE
NCT03948074	Cannabis For Cancer-Related Symptoms (CAFCARS)	Not yet recruiting	Cancer	Interventional	Cannabis/cannabinoids – THC, CBD	Treatment	2	150	2019	Canada	British Columbia Cancer Agency
NCT02983773	Marijuana's Impact on Alcohol Motivation and Consumption	Recruiting	substance use disorder – alcohol	Interventional	Cannabis/cannabinoids – dronabinol	Basic science	2	173	2017	USA	Brown
NCT02492074	Gene-Environment-Interaction: Influence of the COMT Genotype on the Effects of Different Cannabinoids - a PET Study	Not yet recruiting	Healthy	Interventional	Cannabis/cannabinoids – dronabinol, CBD	Basic science	1	60	2020	Germany	Central Institute of Mental Health, Mannheim
NCT03106363	Combined Alcohol and Cannabis Effects on Skills of Young Drivers	Recruiting	Healthy	Interventional	Cannabis/cannabinoids – THC	Other	1	70	2017	Canada	Centre for Addiction and Mental Health
NCT03928015	Evaluation of Dronabinol For Acute Pain Following Traumatic Injury	Not yet recruiting	Pain – post-traumatic pain	Interventional	Cannabis/cannabinoids – dronabinol	Supportive care	2	216	2019	USA	Centura Health
NCT04099355	Investigating the Effect of Dronabinol on Post-surgical Pain	Recruiting	Pain – post-operative pain	Interventional	Cannabis/cannabinoids – dronabinol	Treatment	1	80	2019	USA	Columbia - New York Psychiatric Institute
NCT03775200	The Safety and Efficacy of Psilocybin in Participants With Treatment Resistant Depression (P-TRD)	Recruiting	Depression	Interventional	Psilocybin	Treatment	2	216	2019	UK	COMPASS Pathways
NCT03766269	Dronabinol Opioid Sparing Evaluation (DOSE) Trial	Recruiting	Pain – chronic pain	Interventional	Cannabis/cannabinoids – dronabinol	Treatment	2	280	2018	USA	Daisy Pharma Opioid Venture
NCT01964404	Cannabis, Schizophrenia and Reward: Self-Medication and Agonist Treatment?	Recruiting	Psychosis/schizophrenia	Interventional	Cannabis/cannabinoids – dronabinol	Basic science	1	240	2014	USA	Dartmouth College
NCT04203498	Safety and Effectiveness of Nabiximols Oromucosal Spray as Add-on Therapy in Participants With Spasticity Due to Multiple Sclerosis	Not yet recruiting	degenerative diseases – multiple sclerosis	Interventional	Cannabis/cannabinoids – nabiximols	Treatment	3	446	2020	USA	GW Pharmaceuticals, Inc.
NCT03087201	CANNAbinoids in the Treatment of TICS (CANNA-TICS) (CANNA-TICS)	Recruiting	Multiple – Tourette syndrome, tic disorder	Interventional	Cannabis/cannabinoids – nabiximols	Treatment	3	96	2018	Germany	Hannover Medical School
NCT03380442	Psilocybin and Depression (Psilo101)	Not yet recruiting	Depression	Interventional	Multiple – psilocybin, ketamine	Basic science	2	60	2018	Finland	Helsinki University
NCT03429075	Psilocybin vs Escitalopram for Major Depressive Disorder: Comparative Mechanisms (Psilodep-RCT)	Recruiting	Depression	Interventional	Psilocybin	Treatment	2	50	2019	UK	Imperial College London
NCT04158778	Bristol Imperial MDMA in Alcoholism Study (BIMA)	Active, not recruiting	Substance use disorder – alcohol	Interventional	MDMA	Treatment	1	20	2018	UK	Imperial College London
NCT02792257	Trial of Dronabinol Adjunctive Treatment of Agitation in Alzheimer's Disease (AD) (THC-AD)	Recruiting	Degenerative diseases – Alzheimer's disease	Interventional	Cannabis/cannabinoids – dronabinol	Treatment	2	160	2017	USA	Johns Hopkins University
NCT02145091	Effects of Psilocybin on Behavior, Psychology and Brain Function in Long-term Meditators	Active, not recruiting	Healthy	Interventional	Psilocybin	Basic science	1	100	2014	USA	Johns Hopkins University
NCT02243813	Effects of Psilocybin-facilitated Experience on the Psychology and Effectiveness of Professional Leaders in Religion	Recruiting	Healthy	Interventional	Psilocybin	Basic science	1	12	2015	USA	Johns Hopkins University
NCT03609853	Behavioral Pharmacology of THC and D-limonene	Recruiting	Healthy	Interventional	Cannabis/cannabinoids – THC, limonene (terpene)	Basic science	1	20	2019	USA	Johns Hopkins University
NCT03181529	Effects of Psilocybin in Major Depressive Disorder	Active, not recruiting	Depression	Interventional	Psilocybin	Treatment	2	24	2017	USA	Johns Hopkins University
NCT04052568	Effects of Psilocybin in Anorexia Nervosa	Recruiting	Anorexia nervosa	Interventional	Psilocybin	Treatment	1	18	2019	USA	Johns Hopkins University
NCT04123314	Psilocybin for Depression in People With Mild Cognitive Impairment or Early Alzheimer's Disease	Recruiting	Multiple – depression, depressive symptoms, Alzheimers disease, mild cognitive impairment	Interventional	Psilocybin	Treatment	1	20	2019	USA	Johns Hopkins University
NCT04201197	Interactions Between Cannabinoids and Cytochrome P450-Metabolized Drugs	Not yet recruiting	Healthy	Interventional	Cannabis/cannabinoids – inje cocktail, THC Cannabis extract, THC/CBD cannabis extract	Basic science	1	25	2020	USA	Johns Hopkins University
NCT04130633	Behavioral Pharmacology of THC and Alpha-pinene	Not yet recruiting	Healthy	Interventional	Cannabis/cannabinoids – THC, alpha-pinene (terpene)	Basic science	1	32	2020	USA	Johns Hopkins University
NCT01943994	Psilocybin-facilitated Smoking Cessation Treatment: A Pilot Study	Recruiting	Substance use disorder – nicotine	Interventional	Psilocybin	Treatment	NA	95	2008	USA	Johns Hopkins University
NCT03418714	Effects of Salvinorin A on Brain Function	Active, not recruiting	Healthy	Interventional	Salvinorin A	Other	1 and 2	20	2017	USA	Johns Hopkins University
NCT03555968	Effects of THC and Alcohol on Driving Performance	Not yet recruiting	Healthy	Interventional	Cannabis/cannabinoids – THC	Basic science	4	135	2020	Canada	Lakehead University
NCT03655717	Using Imaging to Assess Effects of THC on Brain Activity (fNIRS)	Recruiting	Substance use disorder – marijuana/cannabis	Interventional	Multiple – dronabinol, ethanol	Diagnostic	4	50	2018	USA	Massachusetts General Hospital
NCT03550352	Cannabinoids in PLWHIV on Effective ART	Not yet recruiting	HIV	Interventional	Cannabis/cannabinoids – THC, CBD	Treatment	2	26	2018	Canada	McGill University
NCT03773796	Nabilone for Non-motor Symptoms in Parkinson's Disease (NMS-Nab2)	Recruiting	Degenerative diseases – Parkinson's disease	Interventional	Cannabis/cannabinoids – nabilone	Treatment	3	48	2018	Austria	Medical University of Innsbruck
NCT04155008	Nutrition and Pharmacological Algorithm for Oncology Patients Study	Not yet recruiting	Cancer	Interventional	Cannabis/cannabinoids – dronabinol	Supportive care	4	30	2019	USA	Montefiore Medical Center
NCT03422861	Nabilone Use For Acute Pain in Inflammatory Bowel Disease Patients	Not yet recruiting	Pain – inflammatory bowel disease	Interventional	Cannabis/cannabinoids – nabilone	Supportive care	NA	80	2019	USA	Mount Sinai Health System
NCT01404754	Psychological Effects of Methylenedioxymethamphetamine (MDMA) When Administered to Healthy Volunteers (MT-1)	Enrolling by invitation	Healthy	Interventional	MDMA	Basic science	1	100	2011	USA	Multidisciplinary Association for Psychedelic Studies (MAPS)
NCT03181763	Evaluation of MDMA on Startle Response	Recruiting	Healthy	Interventional	MDMA	Basic science	1	30	2017	USA	Multidisciplinary Association for Psychedelic Studies (MAPS)
NCT04077437	A Multi-Site Phase 3 Study of MDMA-Assisted Psychotherapy for PTSD II	Not yet recruiting	PTSD	Interventional	MDMA	Treatment	3	100	2020	USA	Multidisciplinary Association for Psychedelic Studies (MAPS)
NCT03537014	A Multi-Site Phase 3 Study of MDMA-Assisted Psychotherapy for PTSD (MAPP1)	Recruiting	PTSD	Interventional	MDMA	Treatment	3	100	2018	USA	Multidisciplinary Association for Psychedelic Studies (MAPS)
NCT03485287	Study of Safety and Effects of MDMA-assisted Psychotherapy for Treatment of PTSD	Active, not recruiting	PTSD	Interventional	MDMA	Treatment	2	5	2018	USA	Multidisciplinary Association for Psychedelic Studies (MAPS)
NCT03282123	Open Label Multi-Site Study of Safety and Effects of MDMA-assisted Psychotherapy for Treatment of PTSD	Active, not recruiting	PTSD	Interventional	MDMA	Treatment	2	38	2017	USA	Multidisciplinary Association for Psychedelic Studies (MAPS)
NCT04073433	Psychological Effects of Methylenedioxymethamphetamine (MDMA) When Administered to Healthy Volunteers (MT-2)	Not yet recruiting	Healthy	Interventional	MDMA	Basic science	1	150	2020	USA	Multidisciplinary Association for Psychedelic Studies (MAPS)
NCT03606538	MDMA in Subjects With Moderate Hepatic Impairment and Subjects With Normal Hepatic Function	Not yet recruiting	Hepatic impairment	Interventional	MDMA	Basic science	1	16	2020	USA	Multidisciplinary Association for Psychedelic Studies (MAPS)
NCT04030169	Open Label Multi-Site Study of Safety and Effects of MDMA-assisted Psychotherapy for Treatment of PTSD With Optional fMRI Sub-Study	Not yet recruiting	PTSD	Interventional	MDMA	Treatment	2	40	2019	Netherlands	Multidisciplinary Association for Psychedelic Studies (MAPS) - Europe
NCT00957359	Psilocybin Cancer Anxiety Study	Active, not recruiting	Cancer	Interventional	Psilocybin	Treatment	1	29	2009	USA	New York University
NCT02421263	The Effects of Psilocybin-Facilitated Experience on the Psychology and Effectiveness of Religious Professionals	Recruiting	Healthy	Interventional	Psilocybin	Health services research	1	12	2015	USA	New York University
NCT02061293	A Double-Blind Trial of Psilocybin-Assisted Treatment of Alcohol Dependence	Recruiting	Substance use disorder – alcohol	Interventional	Psilocybin	Treatment	2	180	2014	USA	New York University
NCT03560934	Tetrahydrocannabinol (THC) and Sleep	Recruiting	Healthy	Interventional	Cannabis/cannabinoids – dronabinol	Basic science	1	14	2018	USA	Oregon Health and Science University
NCT03289949	The Neurobiological Effect of 5-HT2AR Modulation	Recruiting	Healthy	Interventional	Psilocybin	Basic science	1	45	2019	Denmark	Rigshospitalet
NCT03337503	Safety and Efficacy of Medical Cannabis Oil in the Treatment of Patients With Chronic Pain	Recruiting	Pain – chronic pain	Interventional	Cannabis/cannabinoids – THC, CBD	Treatment	4	160	2018	Canada	Sante Cannabis
NCT04060108	Stanford Reward Circuits of the Brain Study - MDMA (RBRAIN-MDMA)	Not yet recruiting	Healthy	Observational	MDMA	NA	NA	40	2020	USA	Stanford University
NCT03646552	A Study to Examine the Efficacy of a Therapeutic THX-110 for Obstructive Sleep Apnea	Recruiting	Obstructive sleep apnea	Interventional	Cannabis/cannabinoids – THX-110 (dronabinol + PEA)	Treatment	2	30	2018	Israel	Therapix Biosciences Ltd.
NCT03651726	A Study to Examine the Efficacy of a Therapeutic THX-110 for Tourette Syndrome	Not yet recruiting	Tourette syndrome	Interventional	Cannabis/Cannabinoids – THX-110 (Dronabinol + PEA)	Treatment	2	60	2018	Israel	Therapix Biosciences Ltd.
NCT02037126	Psilocybin-facilitated Treatment for Cocaine Use	Recruiting	Substance use disorder – cocaine	Interventional	Psilocybin	Treatment	2	40	2015	USA	University of Alabama
NCT03153579	LSD Treatment in Persons Suffering From Anxiety Symptoms in Severe Somatic Diseases or in Psychiatric Anxiety Disorders (LSD-assist)	Recruiting	Anxiety disorders	Interventional	LSD	Treatment	2	40	2017	Switzerland	University Hospital, Basel
NCT03866252	LSD Therapy for Persons Suffering From Major Depression (LAD)	Recruiting	Depression	Interventional	LSD	Treatment	2	60	2019	Switzerland	University Hospital, Basel
NCT03781128	Lysergic Acid Diethylamide (LSD) as Treatment for Cluster Headache (LCH)	Recruiting	Headache disorders – cluster headache	Interventional	LSD	Treatment	2	30	2019	Switzerland	University Hospital, Basel
NCT03527316	Effect of Methylenedioxymethamphetamine (MDMA) (Serotonin Release) on Fear Extinction (MFE)	Recruiting	Healthy	Interventional	MDMA	Basic science	1	30	2019	Switzerland	University Hospital, Basel
NCT03604744	Direct Comparison of Altered States of Consciousness Induced by LSD and Psilocybin (LSD-psilo)	Recruiting	Healthy	Interventional	Multiple – psilocybin, LSD	Basic science	1	30	2019	Switzerland	University Hospital, Basel
NCT03912974	Effects of SERT Inhibition on the Subjective Response to Psilocybin in Healthy Subjects	Recruiting	Healthy	Interventional	Multiple – SSRI (escitalopram), Psilocybin	Basic science	1	24	2019	Switzerland	University Hospital, Basel
NCT03661892	Pilot, Syndros, Decreasing Use of Opioids in Breast Cancer Subjects With Bone Mets	Recruiting	Cancer	Interventional	Cannabis/cannabinoids – dronabinol	Treatment	1	20	2018	USA	University of Arizona
NCT03300947	Psilocybin for Treatment of Obsessive Compulsive Disorder (PSILOCD)	Recruiting	OCD	Interventional	Psilocybin	Treatment	1	15	2019	USA	University of Arizona
NCT02460692	Trial of Dronabinol and Vaporized Cannabis in Neuropathic Low Back Pain	Recruiting	Pain – neuropathic low-back pain	Interventional	Cannabis/cannabinoids – dronabinol, cannabis	Treatment	2	120	2016	USA	University of California, San Diego
NCT02950467	Psilocybin-assisted Group Therapy for Demoralization in Long-term AIDS Survivors	Active, not recruiting	Multiple – distress/grief, depression	Interventional	Psilocybin	Treatment	1	36	2018	USA	University of California, San Francisco
NCT03790618	Effect of Stimulant Drugs on Social Perception (ESP)	Recruiting	Healthy	Interventional	Multiple – MDMA, methamphetamine	Basic science	1	40	2016	USA	University of Chicago
NCT03530800	Dronabinol in Trichotillomania and Other Body Focused Repetitive Behaviors	Recruiting	Trichotillomania	Interventional	Cannabis/cannabinoids – dronabinol	Treatment	2	50	2018	USA	University of Chicago
NCT03809546	Individual Differences in Drug Response (IDT)	Recruiting	Healthy	Interventional	Cannabis/cannabinoids – dronabinol	Basic science	1	60	2018	USA	University of Chicago
NCT03790358	Mood Effects of Serotonin Agonists	Recruiting	Healthy	Interventional	LSD	Basic science	1	40	2018	USA	University of Chicago
NCT04053036	Effects of Drugs on Responses to Brain and Emotional Processes (MAT)	Recruiting	Autism spectrum disorder	Interventional	MDMA	Basic science	1	45	2019	USA	University of Chicago
NCT03944954	Neural Mechanisms of Cannabinoid-impaired Decision-Making in Emerging Adults	Recruiting	Substance use disorder – marijuana/cannabis	Interventional	Cannabis/cannabinoids – dronabinol	Basic science	1	40	2017	USA	University of Kentucky
NCT03380728	Ibogaine in the Treatment of Alcoholism: a Randomized, Double-blind, Placebo-controlled, Escalating-dose, Phase 2 Trial	Not yet recruiting	Substance use disorder – alcohol	Interventional	Ibogaine hydrochloride	Treatment	2	12	2020	Brazil	University of Sao Paulo
NCT03744091	Evaluation of the Pharmacokinetics of Prana P1 Capsules	Active, not recruiting	Healthy	Interventional	Cannabis/cannabinoids – THC	Treatment	1	13	2018	West Indies	University of the West Indies
NCT03215940	Treatment of Chronic Pain With Cannabidiol (CBD) and Delta-9-tetrahydrocannabinol (THC)	Recruiting	Pain – chronic pain	Interventional	Cannabis/cannabinoids – dronabinol, CBD	Treatment	1	75	2018	USA	University of Utah
NCT04161066	Adjunctive Effects of Psilocybin and Buprenorphine	Not yet recruiting	Substance use disorder – opioids	Interventional	Psilocybin	Health services research	1	10	2020	USA	University of Wisconsin
NCT03715127	Clinical, Neurocognitive, and Emotional Effects of Psilocybin in Depressed Patients - Proof of Concept	Recruiting	Depression	Interventional	Psilocybin	Treatment	2	60	2019	Switzerland	University of Zurich
NCT03736980	Beyond the Self and Back: Neuropharmacological Mechanisms Underlying the Dissolution of the Self	Active, not recruiting	Healthy	Interventional	Psilocybin	Basic science	NA	140	2018	Switzerland	University of Zurich
NCT03853577	Characterization of Altered Waking States of Consciousness in Healthy Humans	Recruiting	Healthy	Interventional	Psilocybin	Basic science	NA	25	2019	Switzerland	University of Zurich
NCT04141501	Clinical and Mechanistic Effects of Psilocybin in Alcohol Addicted Patients	Not yet recruiting	Substance use disorder – alcohol	Interventional	Psilocybin	Treatment	2	60	2020	Switzerland	University of Zurich
NCT03866174	A Study of Psilocybin for Major Depressive Disorder (MDD)	Recruiting	Depression	Interventional	Psilocybin	Treatment	2	80	2019	USA	Usona Institute
NCT03008005	Effects of Delta-9 Tetrahydrocannabinol (THC) on Retention of Memory for Fear Extinction Learning in PTSD: R61 Study	Recruiting	PTSD	Interventional	Cannabis/cannabinoids – dronabinol	Basic science	4	104	2017	USA	Wayne State University
NCT02069366	Cannabinoid Control of Fear Extinction Neural Circuits in Post-traumatic Stress Disorder	Active, not recruiting	PTSD	Interventional	Cannabis/cannabinoids – dronabinol	Basic science	NA	130	2014	USA	Wayne State University
NCT04080427	Effects of Delta9-tetrahydrocannabinol (THC) on Retention of Memory for Fear Extinction Learning in PTSD: R33 Study	Not yet recruiting	PTSD	Interventional	Cannabis/cannabinoids – dronabinol	Basic science	1	100	2020	USA	Wayne State University
NCT04040582	Psychedelics and Wellness Study (PAWS)	Recruiting	Multiple – depression, anxiety, PTSD	Observational	Multiple – All psychedelics	Treatment	NA	5000	2019	USA	Wild 5 Wellness
NCT00678730	Pharmacogenetics of Cannabinoid Response	Active, not recruiting	Healthy	Interventional	Cannabis/cannabinoids – THC	Basic science	1	162	2007	USA	Yale University
NCT00982982	Effects of Delta-9-THC and Iomazenil in Healthy Humans	Active, not recruiting	Psychosis/schizophrenia	Interventional	Cannabis/cannabinoids – THC	Basic science	1	60	2009	USA	Yale University
NCT00700596	Effects of Salvinorin A in Healthy Controls	Active, not recruiting	Healthy	Interventional	Salvinorin A	Basic science	1	41	2009	USA	Yale University
NCT01180374	The Effects of Cannabidiol and ∆-9-THC in Humans	Active, not recruiting	Healthy	Interventional	Cannabis/cannabinoids – dronabinol, CBD	Basic science	1	75	2010	USA	Yale University
NCT01591629	The Effects of ∆-9-THC and Naloxone in Humans	Active, not recruiting	Healthy	Interventional	Cannabis/cannabinoids – THC	Basic science	1	56	2011	USA	Yale University
NCT02335060	N-acetylcysteine Effects on Tetrahydrocannabinol	Active, not recruiting	Healthy	Interventional	Cannabis/cannabinoids – THC	Basic science	1	36	2014	USA	Yale University
NCT02781519	Gender Related Differences in the Acute Effects of Delta-9-Tetrahydrocannabinol in Healthy Humans (THC-Gender)	Recruiting	Healthy	Interventional	Cannabis/cannabinoids – THC	Basic science	1	100	2015	USA	Yale University
NCT02811939	Testing the Interactive Effects of Delta-9-Tetrahydrocannabinol and Pregnenolone: Sub-Study I (THC-PREG-I)	Recruiting	Healthy	Interventional	Cannabis/cannabinoids – dronabinol	Basic science	1	19	2016	USA	Yale University
NCT02811510	Gender Related Differences in the Acute Effects of Delta-9-Tetrahydrocannabinol in Healthy Humans: Sub-Study I (THC-Gender-I)	Recruiting	Healthy	Interventional	Cannabis/cannabinoids – dronabinol	Basic science	1	40	2016	USA	Yale University
NCT02981173	Psilocybin for the Treatment of Cluster Headache	Recruiting	Headache disorders – cluster headache	Interventional	Psilocybin	Treatment	1	24	2016	USA	Yale University
NCT03206463	Cognitive and Psychophysiological Effects of Delta-9-Tetrahydrocannabinol in Bipolar Disorder (THC-BD)	Active, not recruiting	Bipolar disorder	Interventional	Cannabis/cannabinoids – THC	Treatment	1	40	2017	USA	Yale University
NCT03191084	Examine the Feasibility of a Standardized Field Test for Marijuana Impairment: Laboratory Evaluations	Recruiting	Substance use disorder – marijuana/cannabis	Interventional	Cannabis/cannabinoids – THC	Other	1	28	2017	USA	Yale University
NCT03341689	Psilocybin for the Treatment of Migraine Headache	Recruiting	Headache disorders – migraine	Interventional	Psilocybin	Treatment	1	24	2017	USA	Yale University
NCT02102113	Probing the Cannabinoid System in Individuals With a Family History of Psychosis	Active, not recruiting	Psychosis/schizophrenia	Interventional	Cannabis/cannabinoids – THC	Other	NA	21	2014	USA	Yale University
NCT02757313	Neuroscience of Marijuana Impaired Driving (MJDriving)	Recruiting	Substance use disorder – marijuana/cannabis	Interventional	Cannabis/cannabinoids – THC	Other	NA	96	2016	USA	Yale University
NCT03554174	Psilocybin - Induced Neuroplasticity in the Treatment of Major Depressive Disorder	Recruiting	Depression	Interventional	Psilocybin	Treatment	1	18	2018	USA	Yale University
NCT03356483	Efficacy of Psilocybin in OCD: a Double-Blind, Placebo-Controlled Study	Recruiting	OCD	Interventional	Psilocybin	Treatment	1	30	2018	USA	Yale University
NCT03978156	Dronabinol for Pain and Inflammation in Adults Living With Sickle Cell Disease	Recruiting	Pain – sickle cell disease	Interventional	Cannabis/cannabinoids – dronabinol	Treatment	1	30	2019	USA	Yale University
NCT04025359	Effects of Dronabinol in Opioid Maintained Patients (THC)	Recruiting	Substance use disorder – opioids	Interventional	Cannabis/cannabinoids – dronabinol	Treatment	1	20	2019	USA	Yale University
NCT03752918	The Effects of MDMA on Prefrontal and Amygdala Activation in PTSD	Recruiting	PTSD	Interventional	MDMA	Treatment	1	20	2019	USA	Yale University
NCT04199468	THC and Ketamine Effects in Humans: Relation to Neural Oscillations and Psychosis	Recruiting	Healthy	Interventional	Multiple – THC, ketamine	Basic science	1	21	2019	USA	Yale University
NCT03806985	Effects of Psilocybin in Post-Traumatic Headache	Recruiting	Headache disorders – post-traumatic headache	Interventional	Psilocybin	Treatment	1	24	2019	USA	Yale University
NCT02710331	Ethanol and Cannabinoid Effects on Simulated Driving and Related Cognition: Substudy III (THC-ETOH-III)	Recruiting	Healthy	Interventional	Cannabis/cannabinoids – dronabinol	Basic science	1	40	2020	USA	Yale University
